# An asteroidal origin for water in the Moon

**DOI:** 10.1038/ncomms11684

**Published:** 2016-05-31

**Authors:** Jessica J. Barnes, David A. Kring, Romain Tartèse, Ian A. Franchi, Mahesh Anand, Sara S. Russell

**Affiliations:** 1 Department of Physical Sciences, The Open University, Walton Hall, Milton Keynes MK7 6AA, UK; 2 Lunar and Planetary Institute, 3600 Bay Area Boulevard, Houston, Texas 77058, USA; 3 Institut de Minéralogie, de Physique des Matériaux et de Cosmochimie (IMPMC), Muséum National d'Histoire Naturelle, Sorbonne Universités, CNRS, UMPC & IRD, Paris 75005, France; 4 Earth Sciences Department, Natural History Museum, Cromwell Road, London SW7 5BD, UK

## Abstract

The Apollo-derived tenet of an anhydrous Moon has been contested following measurement of water in several lunar samples that require water to be present in the lunar interior. However, significant uncertainties exist regarding the flux, sources and timing of water delivery to the Moon. Here we address those fundamental issues by constraining the mass of water accreted to the Moon and modelling the relative proportions of asteroidal and cometary sources for water that are consistent with measured isotopic compositions of lunar samples. We determine that a combination of carbonaceous chondrite-type materials were responsible for the majority of water (and nitrogen) delivered to the Earth–Moon system. Crucially, we conclude that comets containing water enriched in deuterium contributed significantly <20% of the water in the Moon. Therefore, our work places important constraints on the types of objects impacting the Moon ∼4.5–4.3 billion years ago and on the origin of water in the inner Solar System.

## Abstract (web summary)

Recent samples have shown that the Moon's interior, previously thought to be anhydrous, contains water, yet how this water was delivered is unclear. Here, using isotopic analyses and modelling, Barnes *et al*. show that carbonaceous chondrite-type objects delivered >80% of the Moon's bulk water.

## Graphical abstract

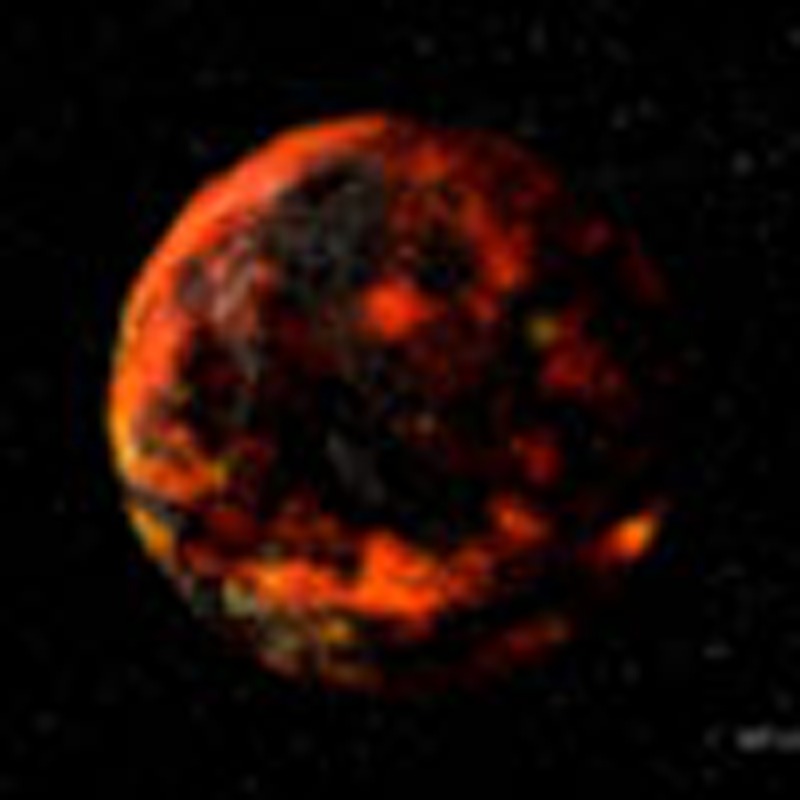

Lunar sample studies have focused on the measurement of H (reported as equivalent amounts of OH or H_2_O) in lunar volcanic glass beads^1^,^2^,^3^, melt inclusions^1^,^2^,^4^ and apatite in lunar basalts and highlands samples^5^,^6^,^7^,^8^. From this compilation of work the bulk silicate Moon (BSM) is estimated to contain between ∼10 and 300 p.p.m. H_2_O^4,9,10^. A few of these studies reported the identification of a primordial H-isotopic signature^2^,^7^,^8^, corresponding to Earth-like δD values between ∼−200 and ∼+180‰ (where δ*D=*[(*D/H*)_sample_/(*D/H*)_SMOW_—*1*] × 1,000; SMOW: standard mean ocean water). Therefore, the similar nature of water (the term water is hereafter used to refer to H_2_O equivalent) in the Earth and Moon suggests that water locked up inside of the Moon was either inherited directly from the proto-Earth or delivered to the Earth–Moon system shortly after the formation of the Moon (Fig. 1) through impacting carbonaceous chondrite (CC)-type asteroids, since bulk D/H ratios for most CC-types^11^ are consistent with those of terrestrial^12^,^13^ and lunar water (Fig. 2).

Models for the differentiation of the Moon invoke the presence of a lunar magma ocean (LMO) and place the lifetime of this LMO to between ∼10 and 200 Ma after the Moon-forming event (as reviewed by Elkins-Tanton *et al*.^14^), which necessitates that volatile accretion occurred relatively early in the Moon's geological history (Fig. 1). The final dregs of the LMO, enriched in incompatible elements such as potassium (K), rare-earth-elements (REEs) and phosphorous (P)—collectively termed urKREEP—should have contained the vast majority of the lunar water, as OH would have been incompatible in the crystallizing mantle cumulates and the crust^10^. Lunar samples with a significant KREEP component, such as Mg-suite rocks and KREEP basalts^7^,^8^, are characterized by H-isotopic compositions that are similar to those estimated for the mantle source regions of picritic glasses and mare basalts^2^,^3^,^6^, suggesting that the crystallization of the LMO did not result in significant fractionation of the H isotopes of water. To retain volatiles in the LMO, a conductive lid is required, to have prevented significant loss of water via degassing of H-bearing species, and is consistent with the formation of an early anorthosite crust a few kilometres thick in the first few thousand years of the Moon's history^14^. It is likely that the lunar crust then grew by continuous underplating of more anorthositic materials over a period of between ∼10 and ∼200 million years until the LMO completely solidified, leading to the mantle cumulate overturn (see review by Elkins-Tanton *et al*.^14^). This time period between lunar accretion and the overturn of mantle cumulates permits any water (and other volatiles) accreted to the LMO to be incorporated into the mantle source regions of mare basalts and picritic glasses (>∼100 km deep^15^), and the urKREEP liquid provided that impactors were able to breach the lunar crust. Crystallization of an insulating crust likely started only after ∼80% LMO crystallization^14^. During its initial growth the crust was likely hot and tenuous and perhaps even re-melted if tidal heating played an important role^14^, meaning not only that impactors could have reached the LMO melt pool but also that many early impact structures could have been erased during crustal underplating and modification. Therefore, volatile delivery through the earliest lunar crust into the LMO could have balanced out any potential loss of volatiles (accreted or indigenous) by degassing (before 80% solidification).

Studies that have modelled the flux of impactors to the Moon posit that it was likely that the time period between lunar accretion and differentiation received relatively fewer impact events than during the late heavy bombardment (LHB) (for example, Morbidelli *et al*.^16^), but importantly this was not a quiescent time. Indeed, there are ∼12 preserved Pre-Nectarian lunar basins that have excavations depths around or greater than 34–45 km (ref. 17), which corresponds to the current estimates for lunar crustal thickness based on observations by the GRAIL spacecraft^18^. This demonstrates that large impacts occurred early in the Moon's history (albeit infrequently compared with the LHB epoch^19^). In summary, the time interval for water delivery to the LMO, hereafter referred to as the late accretion window ‘LAW', is conceptually restricted to between time zero (Moon formation) and up to ∼200 Ma thereafter. Importantly, the bulk of lunar water was delivered during LAW, which is distinct from a much younger basin-forming epoch on the Moon that is commonly referred to as the LHB, lunar cataclysm, or inner Solar System cataclysm^16^,^20^,^21^,^22^.

It has been suggested that water could have been accreted together with the Moon-forming material in the so-called ‘cold start'^9^. Whilst it is plausible that some water may have been retained since the Moon's formation^23^, it is highly unlikely that all of BSM water (up to 300 p.p.m. H_2_O) was accreted in this manner and survived LMO processing. Therefore, we envisage two scenarios that can account for the bulk water inventory of the Moon, the first that water was accreted solely during the LAW and no water was inherited from the Moon-forming material (scenario 1), and the second that a portion (we consider up to 25%) of the BSM water was accreted with Moon-forming materials and was then supplemented by water delivery during the LAW (scenario 2).

In this study we utilize estimates of the water content of the BSM^4^,^9^,^10^ and its H-isotopic composition^2^,^3^,^6^,^7^,^8^ together with the current database for the bulk water contents of chondritic meteorites (Table 1, see Methods section), to determine the amount of water added to the early Moon by impacting objects. We consider a range of possible accretion scenarios during LAW to investigate the implications for the mass of water accreted and populations of accreting objects. This is constrained by the estimates for the proportion of lunar mass that was likely added post-lunar core-formation from studies of highly siderophile elements (HSEs) in lunar basalts (up to 0.02% lunar mass^24^,^25^) and highly volatile elements in mare basalts and picritic glasses (up to 0.4% lunar mass^4^,^9^). Our results indicate that carbonaceous chondrite types of impactors were the most important contributors of water to the lunar interior and that comets (with heavy water, Fig. 2) played a very small role.

## Results

### Mass of asteroidal materials added during the LAW

**Figure 2:**
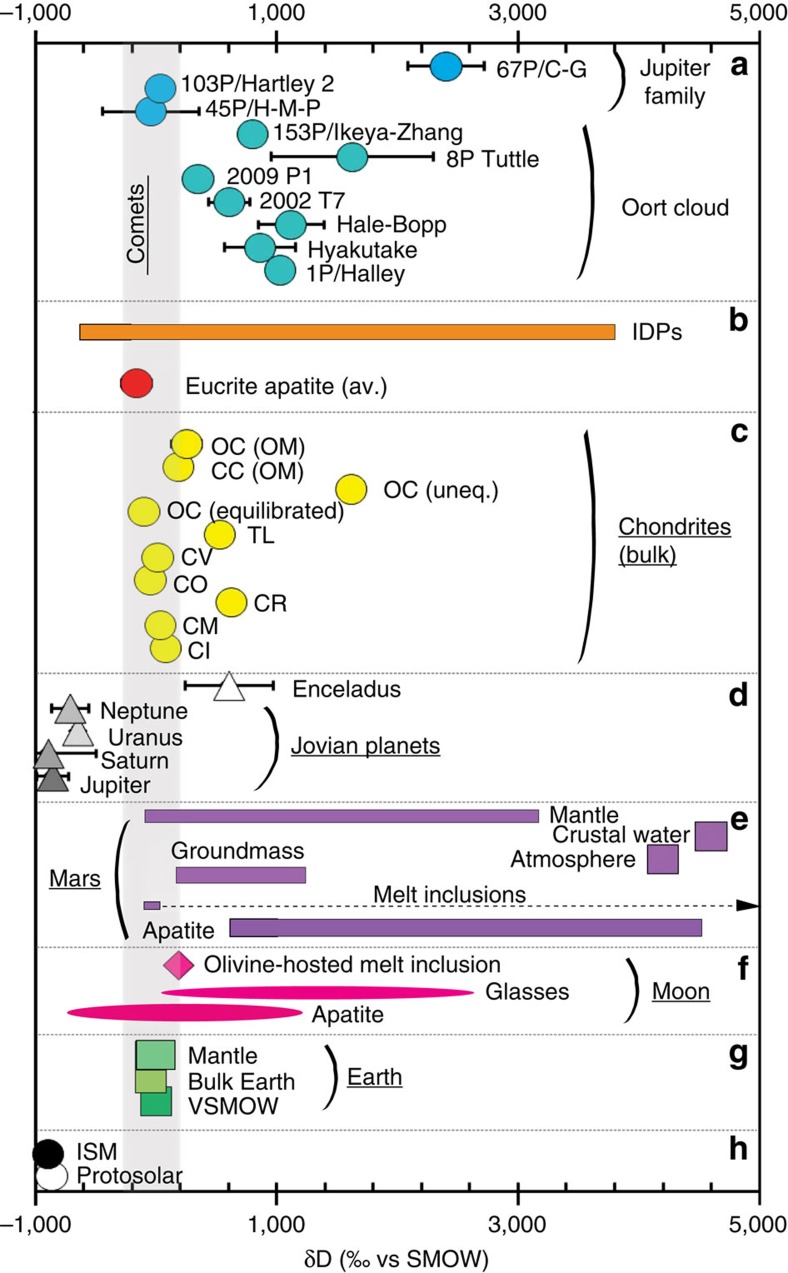
Hydrogen isotope signatures for different objects in the Solar System. The grey bar indicates the range of *δD* values predicted for the lunar interior based on the previous studies of water and its H-isotopic composition in lunar samples^2^,^3^,^6^,^7^,^8^. (**a**) The H-isotopic compositions of comets, where the data for comets 1P/Halley^60^, Hyakutake^61^, Hale-Bopp^62^, C/2002 T7 (ref. 63), C/2009 P1 (ref. 64), 8P/Tuttle^65^, and 153P/Ikeya-Zhang^66^, 45P/Honda-Mrkos-Pajdusakova^57^, 103P/Hartley 2 (ref. 56) and 67P/Churyumov-Gerasimenko^58^. (**b**) The average H-isotopic composition of apatite grains from eucrites^47^, and the range in values for interplanetary dust particles (IDPs)^67^,^68^. (**c**) The bulk H-isotope data for bulk Tagish Lake (TL) and other carbonaceous and ordinary chondrites (CC and OC, respectively)^11^,^55^, carbonaceous and ordinary chondrite hydroxyl and organic matter^54^. (**d**) Hydrogen isotope data for Enceladus^69^ and the jovian planets^70^,^71^,^72^. (**e**) The H-isotopic compositions of martian apatite^73^,^74^,^75^, martian melt inclusions^46^,^74^, martian meteorite groundmass^46^, martian atmosphere^76^ and martian crust and mantle^46^,^73^,^74^,^75^. (**f**) Data for lunar apatite^5^,^6^,^7^,^8^,^33^,^41^,^77^, lunar picritic glasses^2^,^3^ and lunar olivine-hosted melt-inclusions in picritic glass beads^2^. (**g**) The range in H-isotopic compositions of H_2_O on Earth^12^,^13^,^78^. (**h**) The H-isotopic composition of Proto-solar and interstellar medium^42^. For carbonaceous chondrites, CI-, CM-, CV-, CO- and CR- refer to the different groups, named according to one prominent meteorite of the group, respectively Ivuna, Mighei, Vigorano, Ornans and Renazzo. OC stands for ordinary chondrites. Error bars indicate measured analytical uncertainties, please see original references for more information (Encleadus, Jovian planets, and comets error bars, 1 s.d., and Eucrite data, 2 s.d.).

**Figure 3:**
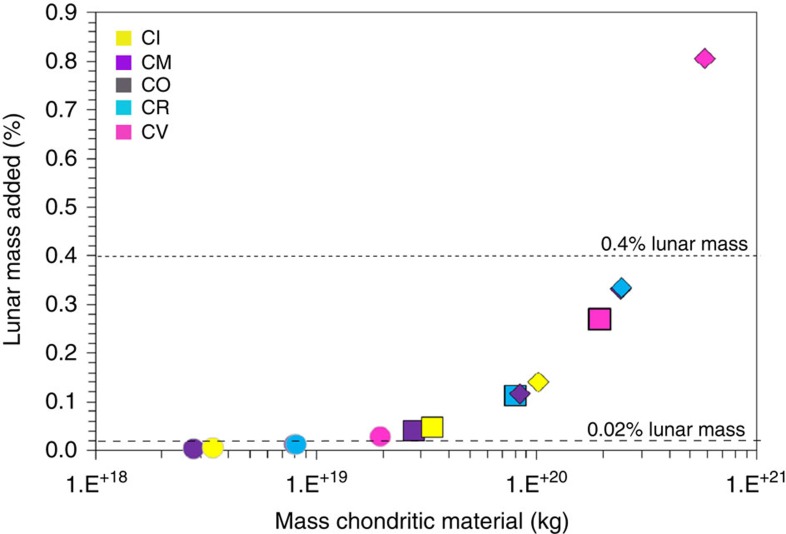
Mass of chondritic material added during late accretion compatible with BSM water estimates. This figure shows the mass of the different types of chondritic material accreted (kg) to the Moon to add 10 (circles), 100 (squares) and 300 p.p.m. H_2_O (diamonds), respectively, to a 400-km-deep LMO, and the corresponding amount in terms of lunar mass. The mass constraints imposed by HSE (0.02% lunar mass added) and highly volatile element abundances (up to 0.4% lunar mass added) are indicated by the dashed lines. Where: CI-type carbonaceous chondrites (CCs) are Ivuna-like, CO are Ornans-like, CV are Vigorano-like, CM are Mighei-like and CR are Renazzo-like, respectively.

Based on the BSM water estimates of McCubbin *et al*.^10^ (∼10–100 p.p.m. H_2_O), and Chen *et al*.^4^ and Hauri *et al*.^9^ (∼100–300 p.p.m. H_2_O), we have calculated the amount of material supplied by chondritic impactors to accrete ∼10, 100 and 300 p.p.m. bulk H_2_O for LMO depths of 400 and 1,000 km (to match the various LMO depths estimated in previous studies^3^,^14^,^26^,^27^), and have recast these estimates into the context of how much lunar mass each would represent (Table 2 and Fig. 3). The types of carbonaceous chondrites are defined in the caption accompanying Fig. 2.

In scenario 1, for example, between 3.94 × 10^16^ kg (∼1 p.p.m. H_2_O) and 2.05 × 10^18^ kg H_2_O (∼52 p.p.m. H_2_O) could have been accreted by CI-type CCs into a 400-km-deep LMO to remain below the addition threshold of <0.02% lunar mass (Table 1). In fact, using this <0.02% lunar mass threshold prohibits the addition of 300 p.p.m. H_2_O by asteroids alone (Table 2). On the other hand, the accretion of up to 0.4% lunar mass in some cases, allows for the addition of H_2_O in amounts greater than the high-end of BSM H_2_O estimates (Table 2).

Importantly, because equilibrated ordinary (OC) and enstatite chondrites (EC) contain very little water (Table 1) the mass of such types of chondritic material added to accrete even 10 p.p.m. H_2_O greatly exceeds the mass constraints imposed by HSE and highly volatile element abundances (Table 2). As a consequence, although ECs are considered an ideal geochemical match for the BSM and the bulk silicate Earth, and Os isotope signatures may suggest a large contribution of OC- and EC-type objects in materials accreted to the Earth–Moon system during late accretion^28^,^29^, it is clear that these types of objects are unlikely to have contributed significant amounts of H_2_O to the lunar interior. The accretion during the LAW of CC material is consistent with the impact of CC-type asteroids during the LHB^20^. HSE data have been interpreted to mean that EC and OC impactors also played an important role during the LHB^30^,^31^, which may reflect a change in impactor populations over the first ∼500 million years of the Moon's evolution and, thus, dynamical mixing of material in the Solar System. The same data have also been interpreted, however, to represent a mixture of OC and CC impactors together with differentiated iron impactors^29^,^30^,^31^,^32^, in which case CC asteroids remained an important source of impactors.

### Assessing the contribution of cometary water during LAW

**Figure 4:**
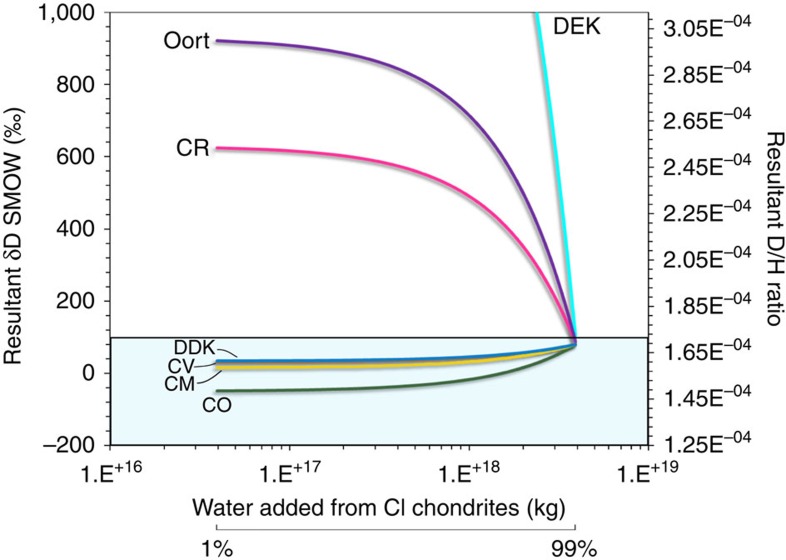
An example of a two-component mixing model for scenario 1 considering that CI type CCs were dominant during the LAW. This model assumes 100 p.p.m. H_2_O in BSM, equivalent to 3.94 × 10^18^ kg of H_2_O for a 400-km-deep LMO. The plot shows the resultant *δD* value and D/H ratios of the water mixture versus the amount of water (kg) supplied by CI-type carbonaceous chondrites as an example (results in Table 3). The bar underneath the x-axis shows how the mass of water is related to % of water mixed. Only final H-isotopic compositions below +100‰ (denoted by the blue box) are acceptable within the constraints of the model (see Methods). Where: CI-type carbonaceous chondrites (CCs) are Ivuna-like, CV are Vigorano-like CO are Ornans-like, CM are Mighei-like and CR are Renazzo-like, respectively. Note that it only takes a couple of per cent contribution of water from Oort or DEK comets to produce H-isotope compositions outside of the model limits. DDK, deuterium-depleted Kuiper belt comets; DEK, deuterium-enriched Kuiper belt comets; Oort, average H-isotopic composition of Oort cloud comets.

The first scenario in this study assumes that the Moon accreted completely dry or that any primordial water was completely lost very early in the Moon's history, and that late-accretion delivered all of the water in the BSM. Considering a BSM of 100 p.p.m. H_2_O (the most recent BSM water estimates overlapping at this abundance^4^,^9^,^10^) and a LMO depth of 400 km (see Methods section), a simple two-component mixing model (equation 1) permits a number of combinations for the mixing of water from bulk CCs (CI, CO, CV, CM type) and deuterium-depleted Kuiper belt (DDK) comets that all result in bulk BSM water *δD* values between ∼−50 and ∼+80‰ (Fig. 4), consistent with existing isotopic limits^2^,^3^,^6^,^7^,^8^ (see Methods).


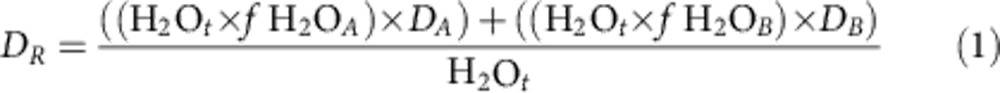


Where; *D*_*R*_ is the resultant D/H ratio, H_2_O_*t*_ refers to the total mass of water added (kg), *f*H_2_O is the proportion (fraction of total) of water accreted by a given type of impactor denoted with an *A* or *B*, characterized by D/H ratios *D*_*A*_ or *D*_*B*_, respectively. The resultant isotopic ratios were subsequently converted into delta notation to ease comparisons.

However, because cometary water, excluding comets 103P and 45P, has significantly elevated *δD* values compared with chondrites (see Methods section), the amount of cometary water that can be added to the lunar interior is far more restricted. In such cases, ∼2% H_2_O from Oort cloud comets can be mixed with 98% H_2_O derived from CI-chondrites, or ∼1% H_2_O is allowed from D-enriched Kuiper belt (DEK) comets (Table 3). Mixing of cometary water with predominantly CV-, CM- or CO-type CCs, respectively, also result in minor cometary contributions of ∼6% for DEK comets and ∼15% from Oort cloud-like objects (Fig. 4 and Table 3).

In scenario 2, we consider that the Moon accreted with between 1 and 25% of BSM H_2_O, with an initial *δD* value of −200‰ (refs 2, 3, 6, 7, 8), and reached a final BSM water content of 100 p.p.m. H_2_O (in agreement with the constraints imposed by modelling of Elkins-Tanton and Grove^26^) (equation (2)). Note that the lower the initial *δD* value is, the higher the maximum possible amount of H_2_O added from a cometary source.


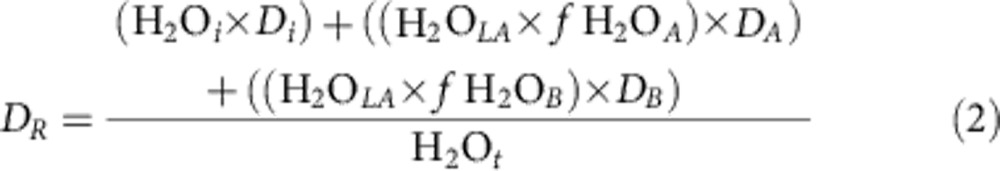


Where; H_2_O_*i*_ refers to the initial mass of water present in the LMO (kg), which varied between 1% and 25% of the total amount of water (H_2_O_*t*_), *D*_*i*_ refers to the initial H-isotopic composition of the water in the LMO, H_2_O_*LA*_ refers to the mass of water added during LAW (kg), *f*H_2_O is the proportion (fraction of amount added during LAW) of water accreted by a given type of impactor denoted with A or B, respectively, and all other parameters are the same as in equation (1).

**Figure 5:**
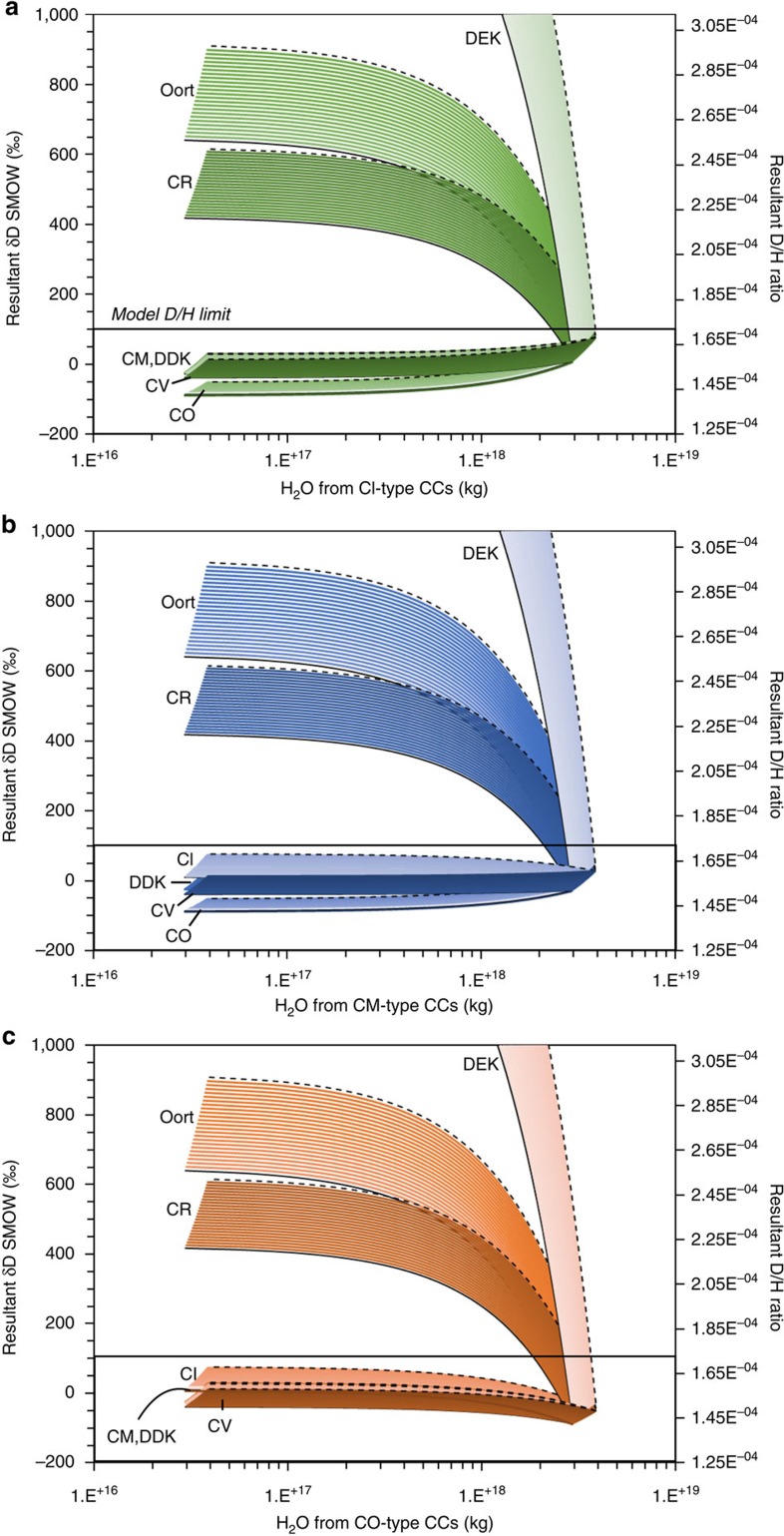
Two-component mixing models for scenario 2 considering that CI or CM or CO-type CCs dominated the impactor population during the LAW. The resultant *δD* value of each water mixture (‰) versus the amount of water (kg) supplied by (**a**) CI-, (**b**) CM- and (**c**) CO-type carbonaceous chondrites. Table 3 also gives results from mixes with CV-type CCs. This model assumes that the LMO initially contained between 1% (dashed black lines) and 25% (solid black lines) of the BSM water (100 p.p.m. H_2_O) with a *δD* value of −200‰. Only final H-isotopic compositions <+100‰ are acceptable within the model constraints (denoted by the black boxes). Where: CI-type carbonaceous chondrites (CCs) are Ivuna-like, CO are Ornans-like, CV are Vigorano-like, CM are Mighei-like and CR are Renazzo-like, respectively.

Mixing of water from CI-type CCs with water from CO, CM, CV-type CCs and DDK comets in different proportions can yield final *δD* values <+100‰ (Table 3), satisfying isotopic constraints^2^,^3^,^6^,^7^,^8^. If deuterium-enriched comets were involved in the addition of water during LAW then up to ∼14% H_2_O could have originated from Oort cloud-type comets and ∼5% H_2_O from DEK comets if the remaining water is assumed to come from CI-type chondrites (Fig. 5a), proportions that increase to ∼7 and ∼19% H_2_O originating from DEK and Oort cloud comets, respectively, if the remaining water came from CM-type chondrites (Fig. 5b and Table 3). Significantly, in the case where CO-type CCs dominated the impactor population (Fig. 5c) up to ∼27% H_2_O could have been contributed by Oort cloud-like comets and up to ∼10% H_2_O from DEK comets (Table 3). In addition, we considered that the LMO underwent some degassing^26^,^33^ before prior to late addition (see Methods section) and found that the results limit the amount of initial water present before late addition (assuming degassed H-isotopic composition of ∼+800‰ (ref. 8)), which further restricts the amount of water contributed by D-rich comets (Table 4).

## Discussion

To a first order, a dominantly asteroidal source of water accreted during the LAW is similar to the dominant source inferred for the subsequent basin-forming epoch of the Moon, based on geochemical^22^,^34^ and mineralogical^20^ markers, implying that asteroids and not comets dominated the impactor population hitting the Moon during its first 500 million years of geological history^35^. Although the source of objects delivered to the Earth–Moon system may have largely remained the same, the dynamical delivery could have been much different, as the basin-forming epoch appears to have been characterized by asteroids with higher impact velocities compared with those during the early accretional epoch (LAW) (Bottke *et al*.^19^ and references therein). We note that the accretion of CC-type material during the LAW, that is required to account for the lunar H-isotope and water abundances in the lunar interior, is distinct from a late veneer dominated by EC- and OC-type objects that has been inferred from Os isotope studies of terrestrial rocks^28^,^29^. This dichotomy could imply that either the largest impactors that preferentially impacted the Earth due to stochastic accretion^19^ were OC- and EC-type objects, while the population of smaller impactors included abundant CC objects that preferentially impacted the Moon, or that while the accreting objects had OC- and EC-type Os isotope signatures, they had CC-type water abundances and H-isotope signatures, consistent with other evidence that support the presence of planetesimal-types that are not represented in modern meteorite falls^36^.

On the basis of combined N isotopes and C/N ratios, the lunar interior seems to be best matched with CO-type CCs^37^. In terms of N, we can estimate the amount of N delivered to the LMO based on our calculations of the mass of chondritic material added during LAW for each chondrite type (Table 2, restricted to consideration of material added to achieve a BSM with ∼100 p.p.m. H_2_O), and the average N contents in the different chondrite types (Table 1). Results yield N contents for the BSM of ∼0.95 to 2.21 p.p.m. for LMO depths of 400 and 1,000 km (Table 5), which is consistent with an average of ∼1 p.p.m. N in the lunar mantle as estimated by Füri *et al*.^38^ If minimal fractionation of ^15^N/^14^N occurred during basalt ascent and eruption^38^,^39^, then the N-isotopic composition measured in mare basalts of between ∼−2 and +27‰ (refs 37, 38) should reflect the isotopic composition of the lunar mantle source regions from which the basalts were derived. If all of the N in the LMO was added during LAW (scenario 1) then mixing of CI-, CM-, CO- and CV-type CCs provide suitable matches for the origin of lunar N (Methods section and Table 1) and restrict cometary contribution of N to as much as ∼5% (Table 5). This is comparable to scenario 2, where considering the lowest mare basalt δ^15^N value of −2 ‰ (ref. 37) to represent the N-isotopic signature of N accreted with Moon-forming materials before LAW, then up to ∼6% N could have been delivered by comets (Table 5). Still using scenario 2 but considering that the isotopic signature of N accreted when the Moon formed was consistent with that of the primordial Earth mantle, which is thought to be as low as ∼−40 ‰ (reviewed by Füri and Marty^40^), increases the possible cometary contribution to a maximum of ∼8% of total lunar interior N.

Therefore, it appears that in order to add the appropriate amounts of H_2_O and N to the lunar interior during LAW, while keeping the H- and N-isotopic compositions in line with sample measurements, requires the contemporaneous accretion of the various types of CCs (mostly CO, CI, CM and CV), together with <∼20% cometary input.

The very-low apparently indigenous lunar *δD* values of ∼−600‰ reported by Robinson *et al*.^41^ are quite anomalous. In fact, such low values have yet to be observed in terrestrial rocks, the lowest *δD* values being ∼−220‰ for Earth's depleted upper mantle^13^. Therefore, such low values reported for an intrusive lunar rock^41^ could potentially indicate the presence of a proto-solar component (*δD* value of ∼−900‰ (ref. 42)) in the lunar mantle, in a similar way that has been used to explain Earth's noble gas record^43^,^44^ and recent data from primitive terrestrial basalts^13^. If this source is indeed required to explain the extremely low H-isotopic compositions observed^41^, then this signature must have been added to the Moon from either material accreted from the proto-Earth or by the impactor involved in the formation of the Moon. Alternatively, such a low *δD* value could have arisen from contamination of a water-poor magma by solar wind-derived H present in the lunar regolith during magma ascent and emplacement.

The mantles of the early-Earth^13^,^45^ the Moon^2^,^3^,^6^,^7^,^8^ and Mars^46^ all seem to be characterized by water with remarkably similar H-isotopic compositions, suggesting that the same types of accreting objects as those modelled here for the Moon delivered a vast majority of the water to the rocky planets in the inner Solar System. Likewise, isotopic analyses of differentiated meteorites^47^ suggest that the interior of the eucrite parent body, probably the asteroid 4-Vesta, contained water characterized by H-isotopic signatures indistinguishable to that of the terrestrial planetary objects in the inner Solar System (Fig. 2).

Regarding the timing of volatile accretion to the Moon, major constraints are imposed by the time interval between lunar accretion and solidification of the LMO and overturn of mantle cumulates (∼10–200 Myr after Moon formation^14^) since there is water in the mantle source regions of the mare basalts^4^,^6^ and picritic glasses^1^,^2^,^3^,^9^. It is possible that some water with Earth-like H-isotopic characteristics may have been accreted with the Moon-forming material, to explain the H-isotopic composition of water in the source regions for the picritic glasses (that were likely deep enough in the lunar mantle to have escaped processes such as degassing of H_2_ (ref. 48)). To account for the H-isotopic composition of the primordial lunar mantle and the urKREEP reservoir together, it is necessary that any initial lunar water was complemented by the continuous accretion of water from CC-type objects to the LMO during LAW. Incidentally, late accretion of material to the Earth–Moon system did not only affect their volatile inventories^49^ but could also explain the recently identified difference between the bulk silicate Earth and BSM in terms of W isotopes^50^,^51^.

In summary, the lunar interior is characterized by an average bulk water content of the order of ∼100 p.p.m. based on samples studies^4^,^9^,^10^ and we propose that the majority of this water was delivered during the *ca*. 10–200 million year-long LMO phase of lunar differentiation, with the possibility that some of this water was inherited proto-Earth-derived materials. This late accretion was dominated by water-rich carbonaceous chondrite asteroids, on the basis of H and N data, likely comprising a mixture of CO-, CI-, CM- and possibly CV-type CCs, with a minor contribution of water from deuterium-rich Oort cloud or Kuiper belt comets (much less than 20% of the total water in the BSM). Our work, therefore, confirms that the inner Solar System received a similar flux of asteroidal and cometary material for much of its early history from 4.5 billion years (this work and Dauphas *et al*.^52^) to at least 3.9 Gyr (ref. 20) (LHB).

## Methods

### Mass balance calculations

For mass balance calculations, we considered a lunar radius of 1,737.4 km, a mantle density of 3,300 kg m^−3^, and varied the depth of the LMO from 400 to 1,000 km. We then calculated the mass of chondritic material added to the lunar interior that would result in BSM water contents of 10, 100 and 300 p.p.m. H_2_O, and considered both the addition of up to 0.02% lunar mass^24^,^25^ and up to 0.4% lunar mass^4^,^9^. Note that for a 400-km-deep LMO, a BSM with 10 p.p.m. H_2_O equates to 3.94 × 10^17^ kg H_2_O, 100 p.p.m. equates to 3.94 × 10^18^ kg H_2_O, and 300 p.p.m. equates to 1.18 × 10^19^ kg H_2_O (6.70 × 10^17^ kg H_2_O, 6.70 × 10^18^ kg H_2_O, and 2.01 × 10^19^ kg H_2_O, for BSM H_2_O contents of 10, 100 and 300 p.p.m., respectively, for a 1,000-km-deep LMO).

Data for the average water contents of different carbonaceous, ordinary and enstatite chondrites that were used in these calculations are shown in Table 1. For carbonaceous chondrites, CI-, CM-, CV-, CO- and CR- refer to the different groups, named according to one prominent meteorite of the group, respectively Ivuna, Mighei, Vigorano, Ornans and Renazzo. OC stands for ordinary chondrites. The results of these calculations, presented in Table 2, allowed us to identify suitable impactors for the modelling presented below. For example, to add 10 p.p.m. H_2_O to the BSM through the accretion of enstatite chondrites alone implies the addition of >1% lunar mass for both 400- and 1,000-km-deep LMO, and, therefore, ECs were not deemed important carriers of water to the lunar interior. The same was also true for equilibrated ordinary chondrites (Table 2). Un-equilibrated ordinary chondrites were also not considered further on the basis of them having prohibitively high H-isotopic compositions (Table 1) for the mixing calculations. Note that all of the scenarios in this work consider that water was efficiently delivered to the Moon regardless of if it originated in asteroids or comets, that is, we have not attempted to calculate the proportions of volatiles lost during impact into the crystallizing LMO.

### Mixing models

In the mixing models, literature data for the water abundances and D/H ratios of bulk carbonaceous chondrite meteorites were utilized. Carbonaceous, ordinary and enstatite chondrite meteorites are characterized by *δD* values mostly between ∼−200 and +300‰ (refs 11, 53, 54, 55). There is also a possibility that the asteroid parent bodies to the CCs may have once contained ice^11^. If the asteroids that delivered water to the Moon contained ice, in addition to their bulk water contents hosted in phyllosilicates and organic matter compounds, then less amounts of chondritic material than those given in Table 2 would have been accreted to the Moon to account for the bulk inventory of water in the lunar interior. Until ice on asteroids is identified and characterized, it remains difficult to quantify how much ice, if any, could have contributed to the mixing of different H-reservoirs within the Moon. Comets 103P/Hartley 2 (ref. 56) and 45P/Honda-Mrkos-Pajdusakova^57^ contain water characterized by *δD* values between ∼+30 and +400‰, whereas other comets studied (for example, 8P/Tuttle and 67P/Churyumov-Gerasimenko) contain water distinctly enriched in D, with *δD* values >+900‰ as reviewed by Hartogh *et al*.^56^ In the modelling, the *δD* value for water in comet 67P/Churyumov-Gerasimenko (+2,400‰ (ref. 58)) was used to represent DEK comets, the *δD* value for water in Oort-cloud comets was set to ∼+930‰ (see Hartogh *et al*.^56^ for a review), and the water *δD* for DDK belt comets was given the same isotopic composition as comet 103P (+34‰ (ref. 56)).

The modelling for scenario 1 assumes no water in the lunar interior before lunar core-formation, and follows equation (1). We used the mid-range estimate for water content for the BSM of ∼100 p.p.m. H_2_O (refs 4, 9, 10) (equivalent to 3.94 × 10^18^ kg of H_2_O for a 400-km-deep LMO) and imposed an upper limit on the resultant *δD* values after mixing of <+100‰ to remain consistent with H-isotopic data obtained for lunar samples^2^,^3^,^6^,^7^,^8^. Only chondrites that were likely to have delivered significant amounts of water to the Moon without adding unrealistic amounts of chondritic material (Table 2) to the LMO were considered in the mixing calculations. Each chondrite-type (CI, CM, CO, CV) was considered in turn as being the most dominant chondrite in the impactor population (Table 3). CR-type CCs were not considered as being a significant part of the impactor population since the bulk *δD* value (Table 1) for such meteorites is significantly elevated with respect to the expected H-isotopic composition of the lunar mantle. In each case, the model extends from having 99% of the accreted water originating from CI-, CM-, CO- or CV-type CCs to having 99% of the water accreted from another type of chondrite or comet.

The two-component mixing models applicable to scenario 2 follow the same constraints as scenario 1 except that they assume that the Moon accreted with some water and late accretion added any remaining water needed to meet the BSM estimate of 100 p.p.m. H_2_O. These calculations follow equation (2) and consider an initial amount of water varying between 1% and 25% of the BSM estimate (that is, 1–25 p.p.m. for a bulk BSM water content of 100 p.p.m. H_2_O) characterized by a *δD* value of −200‰ (refs 2, 3, 6, 7, 8). The model is also adapted to consider each type of CC (CI, CO, CM or CV) as being the dominant source (Table 3).

We also considered that water initially dissolved in the molten LMO might have experienced degassing through loss of H_2_, in a similar way as has been proposed to explain for example the elevated H-isotopic compositions of water (present as OH) measured in apatite in some lunar basalts^6^. Such degassing would have resulted in any remaining water in the LMO before late addition having an elevated *δD* signature of ∼+800‰ (following the work of Tartèse *et al*.^8^). Therefore, we utilized equation (2) and allowed the model for scenario 2 to start with water having a *δD* signature of ∼+800‰ before the late addition of volatiles. All other parameters were kept the same (described above) and from this we were able to model the proportion of water that was initially in the LMO prior to late addition since the model is still constrained to a final H-isotopic composition of <+100‰ and to constrain the maximum contribution made by comets to the water inventory of the Moon assuming an initial H_2_O content of 1 p.p.m. prior to late addition (Table 4).

### Bulk nitrogen content of the Moon and mixing calculations

Using the mass of each type of CC (CI, CO, CM and CV) accreted to an initially dry Moon during LAW (scenario 1) to end up with a BSM containing 100 p.p.m. H_2_O (Table 2), the average N contents for the various types of carbonaceous chondrites (Table 1, data sources^53^,^59^), and assuming LMO depths of 400 or 1,000 km, we have estimated the amounts of N added to an initially N-free LMO, that is, the bulk N content of the BSM (Table 5). Note that in some cases (e.g., CV CCs) the mass of chondritic material added, in order to accrete 100 p.p.m. H_2_O, exceeds the mass balance constraints imposed by HSEs. We then employed the mixing models described above for H_2_O to calculate the acceptable proportion of N contributed by comets (with δ^15^N values ∼+700‰ as a lower limit, see Füri and Marty^40^ and references therein) for scenario 1 (Table 5) and scenario 2 (Table 5), where it was assumed that the initial N-isotopic compositions of N were −2‰ (ref. 37) and −40‰ (Füri and Marty^40^ and references therein) in the same way as was done for H_2_O (Table 3). Notice that like for H_2_O, CR-type chondrites are unsuitable carriers of N due to their elevated δ^15^N values (Table 1).

### Data availability

All relevant data are available from the authors on request and/or are included with the manuscript (in the form of data tables or data within figures).

## Additional information

**How to cite this article:** Barnes, J. J. *et al*. An asteroidal origin for water in the Moon. *Nat. Commun.* 7:11684 doi: 10.1038/ncomms11684 (2016).

## Figures and Tables

**Figure 1:**
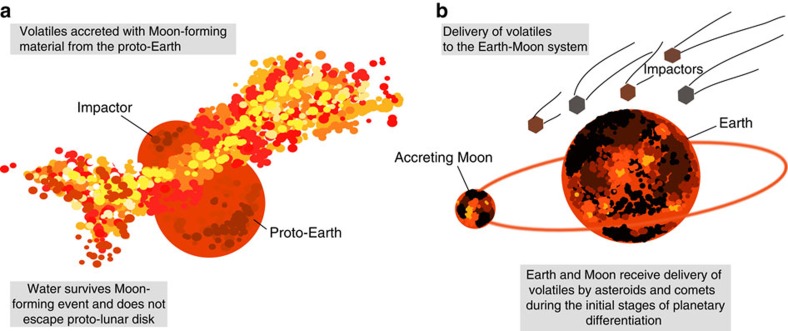
Cartoon showing the possible time windows and scenarios for the accretion of volatiles to the lunar interior. Volatiles were accreted to the Moon during its formation (**a**) and/or continuously delivered by impacting bodies during the *ca.* 10–200 million years of crystallization of the lunar magma ocean (**b**). This graphic is not to scale.

**Table 1:** Data used in mass balance calculations and mixing models.

**Chondrite type**	**Bulk** ***δD*****(‰)**	**s.d.(‰)**	**Bulk H**_**2**_**O(wt.%)**	**s.d.(wt%)**	**Bulk δ**^**15**^**N(‰)**	**s.d.(‰)**	**Bulk N content(wt%)**	**s.d.(wt%)**
CI	80	7	14.05	0.10	47	11	0.31	0.19
CM	30	62	11.57	0.17	32	12	0.11	0.02
CO*	−50	4	4.91	0.09	−9	13	0.05	0.04
CV*	14	8	2.03	0.02	−6	18	0.03	0.02
CR	630	24	4.87	1.44	150	44	0.14	0.07
OC uneq*	1,616	19	1.11	—	—	—	—	—
OC eq	−120	73	0.06	0.07	—	—	—	—
EC	−130	—	<0.05	—	—	—	—	—

CC, carbonaceous chondrite; EC, enstatite chondrites; OC, ordinary chondrites.

Data for bulk H_2_O and bulk H-isotopic compositions of CC and unequilibrated OC meteorites (weighted averages from ref. 11), equilibrated OCs and ECs from ref. 55, and average bulk N and bulk N-isotopic composition^53^,^59^ of chondritic materials. s.d. is the standard deviation of literature data for given chondrite types.

^*^Single data points from Alexander *et al*.^11^ uncertainties represent analytical errors.

**Table 2:** Results of mass balance calculations for the late addition of water to the Moon assuming either a 400- or 1,000-km-deep LMO.

**Chondrite type**	**10 p.p.m. H**_**2**_**O BSM (CC, kg)**	**%LM added**	**100 p.p.m. H**_**2**_**O BSM (CC, kg)**	**%LM added**	**300 p.p.m. H**_**2**_**O BSM (CC, kg)**	**%LM added**	**Max. H**_**2**_**O for 0.02% LM (p.p.m.)**	**Max. H**_**2**_**O for 0.4% LM (p.p.m.)**
400-km-deep LMO								
CI	2.8E+18	<0.01	2.8E+19	0.04	8.4E+19	0.12	52	1,030*
CM	3.4E+18	<0.01	3.4E+19	0.05	1.0E+20	0.14	42	850*
CO	8.0E+18	0.01	8.0E+19	0.11	2.4E+20	0.33	18	360*
CV	1.9E+19	0.03	2.0E+20	0.27	5.8E+20	0.81	7	150
CR	8.1E+18	0.01	8.1E+19	0.11	2.4E+20	0.34	18	358*
EC	7.9E+20	1.09	7.9E+21	10.88	2.4E+22	32.63	<1	<4
OC uneq.	3.6E+19	0.05	3.6E+20	0.49	1.1E+21	1.47	4	81
OC eq.	6.6E+20	0.91	6.6E+21	9.11	2.0E+22	27.32	<1	<5
1,000-km-deep LMO								
CI	4.8E+18	0.01	4.8E+19	0.07	1.4E+20	0.20	30	608*
CM	5.8E+18	0.01	5.8E+19	0.08	1.7E+20	0.24	25	500*
CO	1.4E+19	0.02	1.4E+20	0.19	4.1E+20	0.56	10	213
CV	3.3E+19	0.05	3.3E+20	0.46	9.9E+20	1.37	4	87
CR	1.4E+19	0.02	1.4E+20	0.19	4.1E+20	0.57	10	211
EC	1.3E+21	1.85	1.3E+22	18.47	4.0E+22	55.41	<1	<3
OC uneq.	6.0E+19	0.08	6.1E+20	0.83	1.8E+21	2.50	<3	48
OC eq.	1.1E+21	1.55	1.1E+22	15.46	3.4E+22	46.39	<1	<3

CC, carbonaceous chondrite; EC, enstatite chondrites; HSE, highly siderophile element; HVE, highly volatile element; LMO, lunar magma ocean; OC, ordinary chondrites.

Mass of chondritic material, and corresponding percentage (%) of lunar mass (LM), required for the addition of 10, 100, and 300 p.p.m. H_2_O in 400- and 1,000km-deep LMO during the late accretion window (LAW) for different types of carbonaceous, enstatite and ordinary chondrites. Also included is the maximum amount of water that would be added to the Moon for accretion of 0.02% lunar mass (adhering to HSE abundance constraints^24^,^25^) and for up to 0.4% lunar mass (adhering to the upper limit defined by HVEs^4^,^9^) assuming scenario 1.

^*^Indicates cases where accretion of chondritic material results in H_2_O abundances higher than BSM H_2_O estimates.

**Table 3:** Results from mixing models of scenario 1 and 2.

**Major CC type**	**Scenario 1**		**Scenario 2**			
	**% H**_**2**_**O from DEK comets**	**% H**_**2**_**O from Oort-like comets**	**Max. % H**_**2**_**O from DEK comets LL initial**	**Max. % H**_**2**_**O from DEK comets UL initial**	**Max. % H**_**2**_**O from Oort-like comets LL initial**	**Max. % H**_**2**_**O from Oort-like comets UL initial**
CI	<1	2	1	5	3	14
CM	3	8	3	7	8	19
CO	6	15	6	10	16	27
CV	<4	9	4	8	10	20

Max., maximum; LL initial., lower limit (1% BSM H_2_O) of H_2_O initially in lunar magma ocean; UL initial., upper limit (25% BSM H_2_O) of H_2_O initially in the lunar magma ocean.

Showing the maximum proportions (%) of water permitted from deuterium-enriched Kuiper belt (DEK) and Oort cloud comets assuming the rest of lunar water was delivered by CC-type asteroids.

**Table 4:** Results from the modelling for scenario 2 considering that the water in the LMO before the late addition had undergone significant H_2_ degassing.

**Main CC Type**	**Max. H**_**2**_O in LMO before late accretion (p.p.m.)*	**Max. % H**_**2**_**O added by Oort comets**	**Max. % H**_**2**_**O added by DEK comets**
CI	3	2	0
CM	9	7	3
CO	17	15	6
CV	11	9	4

LMO, lunar magma ocean; max., maximum.

^*^Assuming a BSM of ∼100 p.p.m. H_2_O.

**Table 5:** Results of modelling the amount of N in the lunar interior and the permitted proportion of cometary N added during late accretion.

**Main CC type**	**N content of 400/1,000-km-deep LMO (p.p.m.)**	**Scenario 1: max. % N from comets**	**Scenario 2: max. % N from comets initial δ**^**15**^**N=−2 or −40‰, respectively**
CI	2.21	0.0	0, 0
CO	1.01	∼5.0	6, 8
CM	0.95	0.0	0, 2.5
CV	1.48	4.5	6, ∼8

LMO, lunar magma ocean; max., maximum. N contents based on BSM of 100 p.p.m. H_2_O.
